# AI-driven saliency-guided retinal vessel segmentation framework for sustainable digital pathology

**DOI:** 10.3389/fmed.2026.1801480

**Published:** 2026-04-30

**Authors:** Rajib Guha Thakurta, Mohammed E. Seno, Sami Ahmed Haider, Marwah A. Halwani, Supriya Ashok Bhosale, Mukesh Soni, Masood Ur Rehman

**Affiliations:** 1 School of Computer Science and Applications, REVA University, Bangalore, India; 2 Department of Computer Sciences, College of Sciences, University of Al Maarif, Ramadi, Iraq; 3 Electrical Electronics and Computer Engineer Department, School of Engineering and Physical Sciences, Heriot Watt University, Edinburgh, United Kingdom; 4 Management Information Systems Department, College of Busniess, King Abdulaziz University, Jeddah, Saudi Arabia; 5 Department of Artificial Intelligence, Vishwakarma University, Kondhwa, Pune, India; 6 Division of Research and Development, Lovely Professional University, Phagwara, India; 7 Centre for Research Impact and Outcome, Chitkara University Institute of Engineering and Technology, Chitkara University, Rajpura, Punjab, India; 8 James Watt School of Engineering, University of Glasgow, Glasgow, United Kingdom

**Keywords:** AI-driven sustainable healthcare, boundary refinement, digital pathology, retinal vessel image segmentation, saliency guidance, scale adaptively

## Abstract

**Introduction:**

Accurate segmentation of retinal blood vessels is essential for the early diagnosis of ophthalmic and systemic diseases such as diabetes, hypertension, and cardiovascular disorders. However, challenges such as low contrast, complex vessel geometry, and the presence of pathological artifacts often degrade segmentation performance, particularly for thin vessels and boundary regions.

**Methods:**

To address these challenges, this study proposes an AI-driven saliency-guided boundary refinement framework (SGB-Net). The model integrates a progressive boundary refinement (BR) module to enhance vessel edge representation and a feature-guided encoder-decoder network incorporating scale-adaptive (SA) and attention enhancement (AE) modules. The SA module captures multi-scale contextual features, while the AE module refines feature representations by emphasizing relevant structures and suppressing background noise. The proposed framework was evaluated on three publicly available datasets: DRIVE, STARE, and CHASE_DB1.

**Results:**

Experimental results demonstrate that the proposed method achieves superior segmentation performance, with Dice scores of 98.30%, 78.40%, and 84.60% on the DRIVE, STARE, and CHASE_DB1 datasets, respectively, and AUC values up to 0.9899. The model shows improved capability in preserving thin vessels, enhancing boundary continuity, and reducing false positives under complex imaging conditions compared to existing state-of-the-art methods.

**Discussion:**

The proposed SGB-Net effectively addresses key limitations in retinal vessel segmentation by combining boundary refinement with multi-scale and attention-based feature learning. Its robustness to noise and pathological variations makes it suitable for large-scale digital pathology applications and supports more reliable automated retinal analysis. Future work may focus on improving sensitivity and extending the framework to other medical imaging modalities.

## 1 Introduction

Morphological changes in retinal blood vessels serve as important indicators for hypertension, diabetes, arteriosclerosis, and several ophthalmic and cardiovascular disorders (1). Structural characteristics such as vessel diameter, tortuosity, branching patterns, and crossing behavior provide clinicians with valuable insight into systemic health (2). In practice, however, retinal vessels often exhibit low contrast and visual similarity to the background, resulting in blurred boundaries and partial visibility. These challenges make accurate vessel segmentation essential for reliable computer-aided diagnosis (3–5).

Traditional segmentation approaches—including morphological techniques (6, 7), vessel tracking (8), and matched filtering (9, 10)—offer advantages in simplicity and computational efficiency and do not rely on annotated datasets. Still, their dependence on hand-crafted features based on color, shape, or texture restricts their generalizability, particularly when dealing with diverse illumination, anatomical variations, or complex pathological structures.

Deep learning methods have substantially advanced retinal vessel segmentation by learning hierarchical representations from labeled images. Recent studies have explored residual and multi-scale architectures (11, 12), fusion-based strategies (13), and coarse-to-fine boundary refinement (14). Attention mechanisms have further enhanced vessel localization by highlighting discriminative regions (15). Despite these developments, several unresolved challenges persist: (1) fine vessels and branch terminals are easily lost during down-sampling, leading to broken vessel continuity; (2) blurred edges and low contrast make boundary localization difficult; (3) pathological structures such as hemorrhages or exudates introduce misleading patterns that lead to false positives. These limitations indicate that existing models still struggle to maintain complete vessel topology and accurate boundary representation under complex imaging conditions.

To address these issues, this work presents a saliency-guided boundary refinement framework (SGB-Net) designed to improve the preservation of thin vessels, enhance boundary clarity, and suppress background interference. The model integrates a progressive boundary refinement module with a feature-guided network that incorporates scale-adaptive and attention-based components, allowing complementary extraction of detailed and contextual vessel information. The primary contributions of this work are as follows:

- A progressive boundary refinement module is introduced to strengthen vessel edge representation by integrating low-level detail cues with high-level semantic information.
- A feature-guided network equipped with scale-adaptive and attention-based components is proposed to better capture multi-scale vessel patterns and preserve thin vascular branches.
- A complementary fusion strategy combines boundary and feature-guided representations to improve robustness under varying illumination, noise, and pathological conditions.
- Extensive experiments on public datasets demonstrate improved performance in terms of vessel continuity, thin-vessel segmentation, and boundary precision compared with existing techniques.

Section II presents the proposed framework and its constituent modules. Section III describes the datasets, pre-processing steps, and evaluation metrics. Section IV reports experimental results and comparisons. Section V provides ablation studies and analysis. Finally, Section VI concludes the paper and outlines future research directions.

## 2 Proposed framework

**Figure 1:**
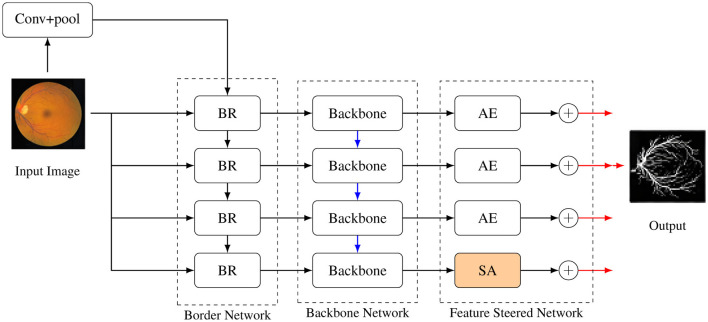
Network architecture with Border Network, Backbone Network, and Feature-Steered Network.

Figure 1 illustrates the comprehensive structure of the proposed network for vessel segmentation in fundus images. The network accepts a raw fundus image as input and forecasts the corresponding outcome. The blue arrows denote down-sampling, the black arrows indicate feature flow, and the red arrows signify up-sampling. BR signifies the boundary refinement module. AE and SA denote the attention enhancement and scale-adaptive modules.

Using ResNet34, which was pre-trained on the ImageNet dataset, as the backbone network helps to decrease parameters and assure accurate segmentation. The fully linked layer and max pooling layer are removed to meet the needs of semantic segmentation and prevent unnecessary data loss. The backbone network streamlines parameter use by producing shared hierarchical features through multiple layers of residual convolutional units; these features are then inputted into the border network and the feature-guided network. To guarantee feature consistency, engineers created the feature-guided network, an encoder-decoder architecture. It detects changes in the topological structure of vessels and mitigates the impact of extraneous information by integrating scale-adaptive (SA) and attention enhancement (AE) modules.

In the boundary network, a boundary refinement module is proposed to solve the problems of unclear and inaccurate target boundaries. By gradually optimizing the boundary features, the boundaries are made more prominent, thereby accurately determining the target boundaries. It should be noted that, due to poor semantic continuity, the boundary network and the feature-guided network do not utilize the bottommost side-output features. Next, each component will be introduced in detail.

### 2.1 Feature-guided network

The feature-guided network is a data-driven encoder–decoder network. It consists of a scale-adaptive (SA) module and an attention enhancement (AE) module. These two modules are responsible for different functions, which help to improve the network's feature extraction and representation ability for input images. The two modules will be introduced in detail below.

#### 2.1.1 Scale-adaptive module

Medical images present large variations in object scale and topology. In retinal fundus images, vessels of the same category may appear with diverse thicknesses and branching patterns. Conventional pyramid-based atrous convolution methods can partially handle scale variation; however, repeated dilation often leads to feature discontinuity and insufficient contextual modeling. To address these issues, a Scale-Adaptive (SA) module is introduced to capture vessel structures at multiple receptive-field scales while preserving global contextual consistency.

The SA module is positioned at the upper stage of the backbone network, where feature maps have lower spatial resolution. Therefore, the use of larger convolution kernels does not introduce excessive computational overhead. Let the backbone output feature map at the final stage be denoted as $\alpha_{4}\in {\mathbb{R}}^{C\times H\times W}$. This feature map serves as the input to the SA module.

The SA module integrates multi-scale convolution, hierarchical feature fusion, and global contextual modeling. Its complete mathematical formulation is described algorithmically in Algorithm 1 Scale-Adaptive Feature Aggregation.

Algorithm 1 Scale-adaptive module.

**Require:** Backbone feature map ***α***_4_
**Ensure:** Scale-adaptive feature representation ***α***_SA_
 1: Apply 5 × 5 convolution on ***α***_4_ to obtain coarse-scale features ***β***_5_
 2: Down-sample ***β***_5_ using max pooling to generate intermediate feature map ***δ***_3_
 3: Apply two successive 3 × 3 convolutions on ***δ***_3_ to extract medium-scale features ***γ***_3_
 4: Down-sample ***γ***_3_ to obtain fine-scale input ***δ***_1_
 5: Apply two successive 1 × 1 convolutions on ***δ***_1_ to extract fine-scale features ***γ***_1_
 6: Refine coarse-scale features by applying a 5 × 5 convolution on ***β***_5_, producing ***γ***_5_
 7: Up-sample ***γ***_1_ repeatedly until its spatial resolution matches ***γ***_3_ and ***γ***_5_
 8: Aggregate multi-scale features by element-wise addition:
 9: ***η***←***γ***_1_+***γ***_3_+***γ***_5_
 10: Apply 1 × 1 convolution on ***α***_4_ to align channel dimensions, yielding ***μ***
 11: Reweight aggregated features via element-wise multiplication:
 12: ***μ***←***μ***⊙***η***
 13: Concatenate ***γ***_1_, ***γ***_3_, ***γ***_5_, and ***μ*** along the channel dimension to form ***κ***
 14: Perform global average pooling on ***α***_4_ to obtain global descriptor ***ρ***_*g*_
 15: Apply 1 × 1 convolution on ***ρ***_*g*_ and up-sample it to match the spatial size of ***κ***, producing ***ρ***
 16: Fuse hierarchical and global contextual features:
 17: ***α***_SA_←***κ***+***ρ***
 18: **return** ***α***_SA_

The Scale-Adaptive Module enhances vessel representation by jointly modeling fine-, medium-, and coarse-scale features while incorporating global contextual cues. This design enables robust segmentation of vessels with varying thickness and complex topology.

#### 2.1.2 Attention enhancement module

**Figure 2:**
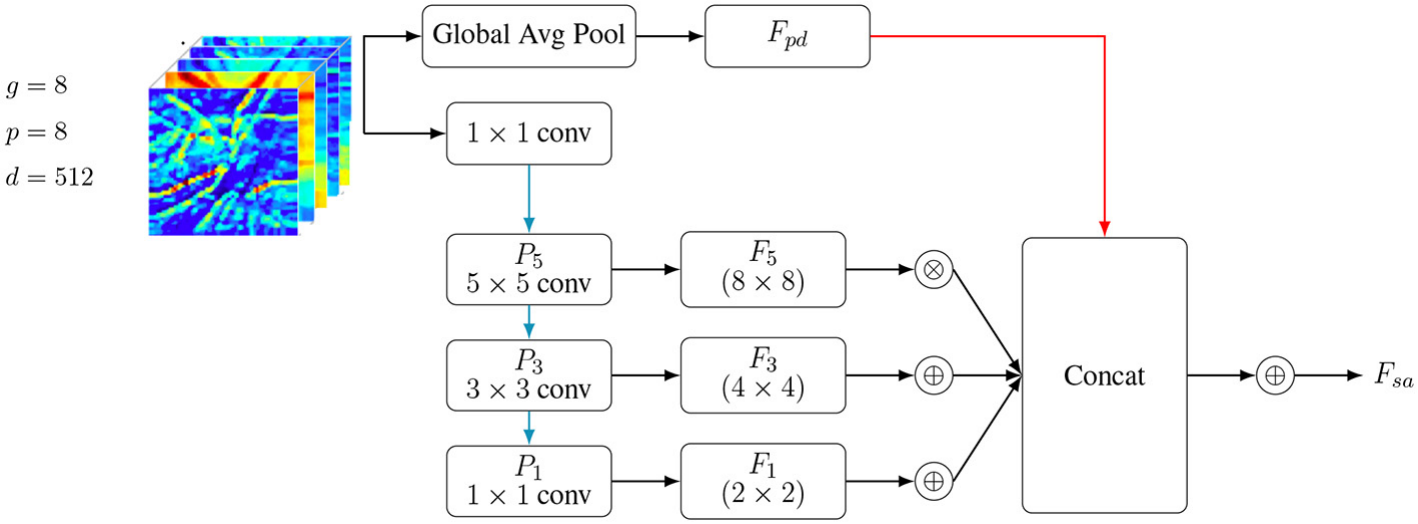
Scale-adaptive module structure (reoriented).

For the decoding module, the scale-adaptive module can provide higher semantic information. Unfortunately, it doesn't help restore the image to its original quality or reduce the impact of complex backdrops. In order to improve image resolution, the CIA-Net decoding module uses linear interpolation as its decoding block (16, 17). Unfortunately, this process may easily generate background noise and remove certain local information inside the image. In contrast, BoxeR (18) and E2EC (19, 20) use complex decoding blocks that demand a lot of computational power. It is well-known that the backbone network's lower levels gather detailed features; yet, insufficient semantic coherence is caused by the lack of contextual information. In contrast, although the resulting geographical information is imperfect, the deeper layers of the backbone network are able to extract detailed abstract properties. The majority of methods include information from nearby stages in a crude, element-by-element manner, ignoring the relationships between contexts. As shown in Figure 2, an AE module is organized as the decoding module to help learn low-level features from high-level ones. This way, it can effectively compensate for detailed information and reduce the impact of cluttered backgrounds.

**Figure 3:**
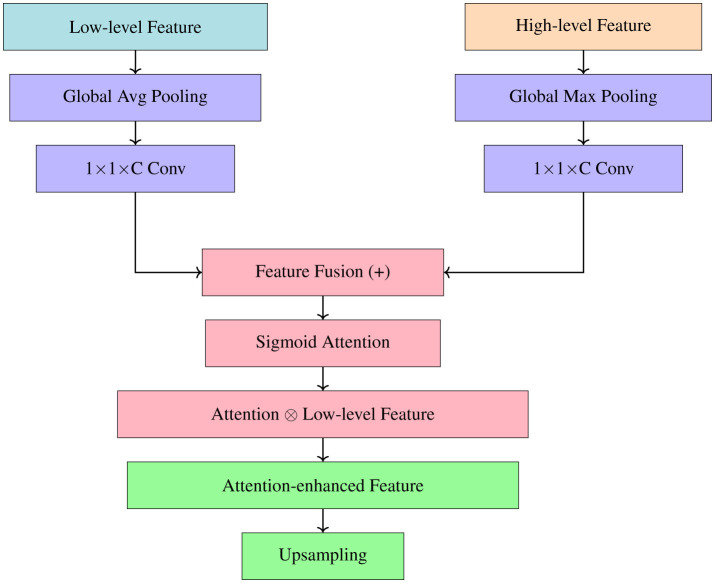
Convolution operations and attention-guided fusion to refine low-level features using high-level semantic information.

To recover precise structural features and efficiently suppress background noise, an Attention Enhancement (AE) module is introduced in the decoding stage. To begin, the decoding path's high-level semantic properties and the backbone network's low-level structure features are subjected to separate global pooling processes, as discussed in Algorithm 2. To decrease the complexity of the parameters and the dimensionality of the channels, the combined features are further subjected to 1 × 1 convolutions. When feature responses need to be readjusted, the Sigmoid activation function is used to create channel-wise attention weights, as shown in Figure 3.

For the low-level backbone features, standard convolution is applied to perform feature dimensionality reduction. These refined low-level features are multiplied with the attention weights to emphasize target regions while suppressing irrelevant background information. Finally, the corrected low-level features and the learned high-level features are fused through pixel-wise addition and progressively up-sampled to restore spatial resolution.

Specifically, the AE module is mathematically formulated as follows.


$$\begin{array}{l} \{\begin{array}{cc} \alpha_{1}^{\ell } & =\delta \left({\text{Conv}}_{1\times 1}\left(\text{Gvp}\left(\beta^{3}\right),\omega_{1\times 1},b_{1\times 1}\right)\right),\\ \alpha_{1}^{h} & =\delta \left({\text{Conv}}_{1\times 1}\left(\text{Gmp}\left(\alpha_{\text{SA}}\right),\omega_{1\times 1},b_{1\times 1}\right)\right),\\ \gamma_{\text{out}}^{1} & =\sigma \left({\text{Conv}}_{1\times 1}\left(\alpha_{1}^{\ell }⊕\alpha_{1}^{h},\omega_{1\times 1},b_{1\times 1}\right)\right)\\ ⊗\delta \left({\text{Conv}}_{3\times 3}\left(\beta^{3},\omega_{3\times 3},b_{3\times 3}\right)\right),\\ \eta_{\text{AE}}^{1} & =\text{up}\left(\gamma_{\text{out}}^{1}⊕\text{up}\left(\alpha_{\text{SA}}\right)\right) \end{array} \end{array}$$ (1)

where δ(·) denotes the ReLU activation function and σ(·) denotes the Sigmoid activation function. $\gamma_{\text{out}}^{1}$ represents the output of the first AE block, and $\eta_{\text{AE}}^{1}$ denotes the fused feature map obtained by combining attention-guided low-level features with scale-adaptive features. Gvp(·) and Gmp(·) denote global average pooling and global max pooling, respectively.

For subsequent AE blocks, the recursive formulation is defined as mention in Equation 2:


$$\begin{array}{l} \{\begin{array}{cc} \alpha_{i}^{\ell } & =\delta \left({\text{Conv}}_{1\times 1}\left(\text{Gvp}\left(\beta^{i-1}\right),\omega_{1\times 1},b_{1\times 1}\right)\right),\\ \alpha_{i}^{h} & =\delta \left({\text{Conv}}_{1\times 1}\left(\text{Gmp}\left(\gamma_{\text{out}}^{i-1}\right),\omega_{1\times 1},b_{1\times 1}\right)\right),\\ \gamma_{\text{out}}^{i} & =\sigma \left({\text{Conv}}_{1\times 1}\left(\alpha_{i}^{\ell }⊕\alpha_{i}^{h},\omega_{1\times 1},b_{1\times 1}\right)\right)\\ ⊗\delta \left({\text{Conv}}_{3\times 3}\left(\beta^{i-1},\omega_{3\times 3},b_{3\times 3}\right)\right),\\ \eta_{\text{AE}}^{i} & =\text{up}\left(\gamma_{\text{out}}^{i}⊕\text{up}\left(\eta_{\text{AE}}^{i-1}\right)\right) \end{array} \end{array}$$ (2)

where $\eta_{\text{AE}}^{i}$ denotes the fused features between two adjacent AE blocks, and *i*∈{2, 3}.

Algorithm 2 Attention enhancement module. 
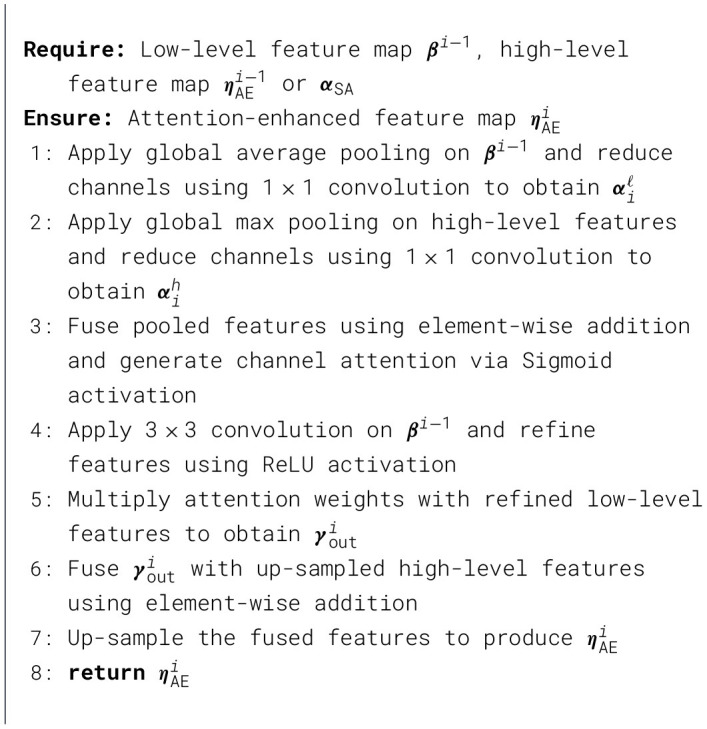

### 2.2 Boundary network

For fundus images with complex and low contrast, the regions of interest are similar in appearance to the surrounding background (such as color and texture), and the proportion of vessel pixels is small. This inter-class similarity makes it difficult to accurately locate the target boundaries and introduces additional false-positive features. Therefore, a boundary refinement module is designed to gradually enlarge the differences between boundary features in a semantically supervised manner. On this basis, a bottom-up boundary network is constructed, adopting a hierarchical refinement structure to achieve feature extraction from coarse to fine, making boundary features easier to distinguish.

As described in Section 2.2, the features retrieved from various layers exhibit differing levels of saliency. Low-level features encompass original detail information, such as color, texture, and boundaries, whereas high-level features pertain to more semantic information, including the shape, structure, and placement of objects. This study necessitates the extraction of border features imbued with greater semantic information to enhance the distinctions between features. The suggested boundary refinement module can encapsulate low-level detail characteristics and high-level semantic details. Furthermore, it compensates for the elimination of original boundary details inside the semantic data. In the chained structure of cascading refinement, coarse attention signals are derived from the detailed aspects of low-level stages and subsequently modified by the semantic properties of high-level stages to produce more precise attention cues, as discussed in Algorithm 3.

Let ***β***_*i*_ denote the side-output feature map extracted from the *i*-th layer of the backbone network, where *i*∈{1, 2, 3}. The initial channel-aligned boundary features are obtained as


$$A\left(\beta_{i}\right)={\text{Conv}}_{1\times 1}\left(\beta_{i},\omega_{1\times 1},b_{1\times 1}\right).$$

An attention map is then generated to emphasize boundary-relevant regions:


$$\tau =\sigma \left(\text{BN}\left({\text{Conv}}_{3\times 3}\left(A\left(\beta_{i}\right),\omega_{3\times 3},b_{3\times 3}\right)\right)\right),$$

where BN(·) denotes batch normalization and σ(·) represents the Sigmoid activation function.

The boundary-refined feature map is computed using a residual refinement strategy:


$$R\left(\beta_{i}\right)={\text{Conv}}_{3\times 3}\left(\tau ,\omega_{3\times 3},b_{3\times 3}\right)+A\left(\beta_{i}\right),$$

where R(·) denotes the refined boundary representation.

For simplicity, the first boundary refinement stage is taken as mention in Equation 3:


$$\begin{array}{l} \{\begin{array}{cc} \gamma_{\text{sum}} & =R\left(\beta^{2}\right)⊕R\left(\beta^{1}\right),\\ \gamma_{y1} & =R\left(\beta^{1}\right)⊗\gamma_{\text{sum}},\\ \gamma_{y2} & =R\left(\beta^{2}\right)⊗\text{down}\left(\gamma_{\text{sum}}\right),\\ \eta_{\text{out}}^{y} & =R\left(\text{Concat}\left\{\gamma_{y1},\gamma_{y2}\right\}\right) \end{array} \end{array}$$ (3)

Here, Concat{·} denotes channel-wise concatenation of hierarchical features from adjacent stages, and $\eta_{\text{out}}^{y}$ represents the refined boundary features produced by the *y*-th stage of the boundary network, where *y*∈{1, 2, 3}.

Finally, the outputs of the boundary network and the feature-guided network are fused and up-sampled, as mention in Equation 4:


$$\begin{array}{l} \text{EQ}=\text{up}\left(\gamma_{\text{out}}^{3}+\eta_{\text{AE}}^{2}+\eta_{\text{out}}^{y}\right). \end{array}$$ (4)

The focal loss function assigns lower weights to easily classified samples to suppress the output of the boundary network, as mention in Equation 5:


$$\begin{array}{l} \ell_{\text{fl}}\left(T_{1}\right)=-{\left(1-S_{1}\right)}^{\gamma }\log T_{1}, \end{array}$$ (5)

where *S*_1_ denotes the predicted probability of positive samples, and γ∈[0, 5] is a modulation factor that emphasizes difficult samples. When γ = 0, the focal loss reduces to the standard cross-entropy loss.

To further address class imbalance, a weighting coefficient λ_*t*_ is introduced, as mention in Equation 6:


$$\begin{array}{l} \ell_{\text{fl}}\left(T_{1}\right)=-\lambda_{t}{\left(1-T_{1}\right)}^{\gamma }\log S_{1}. \end{array}$$ (6)

Algorithm 3 Boundary refinement network. 
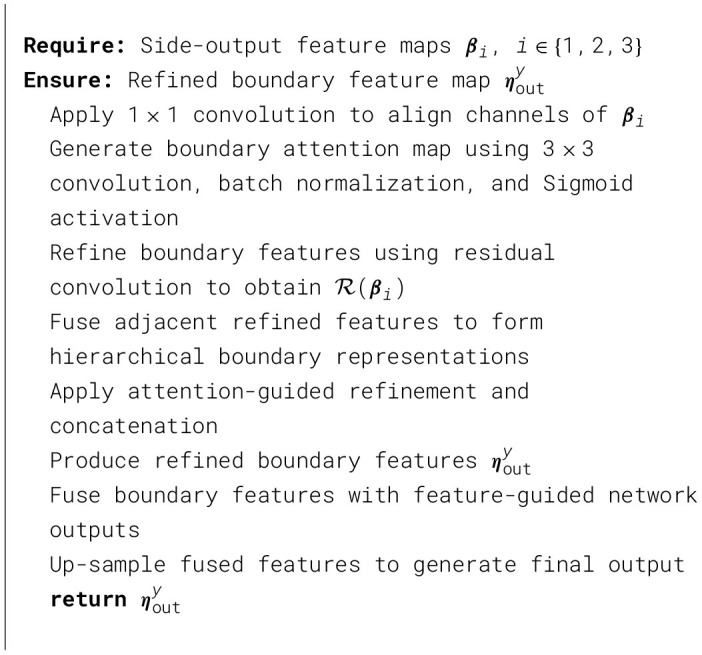

In the experiments, λ_*t*_ and γ are set to 0.25 and 2, respectively. Based on Equation 7 the overall loss of the proposed SGB-Net is defined as:


$$\begin{array}{l} \ell =\ell_{\text{tl}}\left(T_{0}\right)+\eta \ell_{\text{fl}}\left(T_{1}\right)+\psi \ell_{\text{tl}}\left(T_{2}\right), \end{array}$$ (7)

where η and ψ are weighting coefficients that balance the contributions of the boundary network and feature-guided network. Experimental results indicate that the optimal performance is achieved when η = 0.7 and ψ = 0.3. It can be seen from Table 1 that when η and ψ are 0.7 and 0.3, respectively, the segmentation performance of the proposed method reaches the optimal state.

**Table 1:** Segmentation performance under different weighting coefficients.

η	ψ	IoU / %	Dice / %
0.3	0.7	96.48	98.21
0.4	0.6	96.46	98.05
0.5	0.5	96.53	98.13
0.6	0.4	96.41	98.16
0.7	0.3	96.65	98.30

## 3 Experimental data and evaluation metrics

### 3.1 Dataset

In the experiments, five publicly available medical datasets were used, comprising three fundus retinal channel datasets (namely, DRIVE, CHASE DB1, and STARE) and two non-fundus retinal channel datasets (specifically, RIMONE R1 and the EM dataset). The detailed information is as follows:

The DRIVE dataset has 40 color photos at a resolution of 768 by 584 pixels. Each image is accompanied by its respective retinal vessel annotation, comprising 33 healthy fundus retinal channel images and seven photos exhibiting mild diabetic retinopathy.

The CHASE_DB1 dataset comprises 28 high-resolution color pictures obtained from the left and right eyes of 14 individuals. Each image was individually captioned by two separate, competent medical professionals, and the resolution is 999 × 960 pixels.

The STARE dataset comprises 20 color fundus images, each having a resolution of 700 × 605 pixels, consisting of 10 normal and 10 diseased fundus retinal vascular images.

### 3.2 Comparison methods

To evaluate the effectiveness of the proposed SGB-Net, comparisons were conducted with different segmentation methods on three different fundus retinal vessel datasets, including unsupervised methods ACMs (21), Iterative (22), LAD-OS (23), and COSFIRE (24); and supervised methods CPFNet (25), CE-Net (20), KiU-Net (23), MALUNet (24), Bridge-Net (25), DUNet (26), LANet (26), MRUNet (27), UNet++ (28), U-Net (29), MEW-UNet (30), DCUNet (31), HAnet (32), SegFormer (33), LadderNet (34), and AA-UNet (35). The network structures of all comparison methods were directly provided by the authors. To conduct a fair comparison, different comparison methods were retrained, and the remaining parameters were kept consistent with the proposed SGB-Net, aiming to obtain the best results of the comparison methods.

### 3.3 Evaluation metrics

Selected metrics for assessing the level of agreement between segmentation results and the gold standard were sensitivity (Sen), specificity (Spe), accuracy (Acc), area under the receiver operating characteristic curve (AUC), precision-recall (PR) curve, and the F1-score/Dice metric. These measures were used to assess the proposed method's performance on various datasets.

### 3.4 Data pre-processing and patch processing

**Figure 4:**
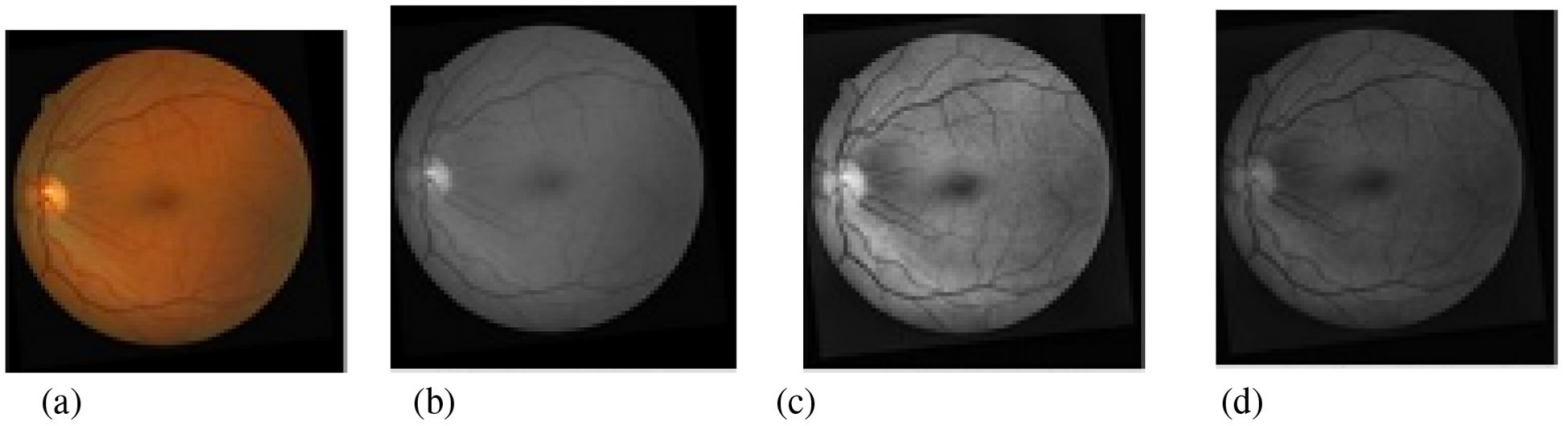
Pre-processing steps of retinal fundus images. **(a)** Original Image. **(b)** Grayscale Image. **(c)** Histogram Equalization. **(d)** Gamma Correction.

**Figure 5:**
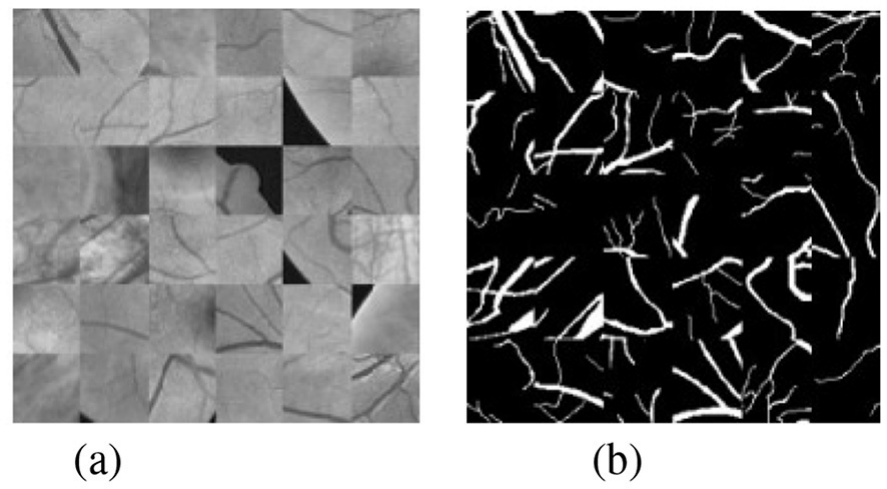
Image patches and their corresponding blood vessel distribution. **(a)** Image block. **(b)** Label.

Figure 4 shows different pre-processing methods. First, the original images are converted into single-channel grayscale images with higher contrast, and then histogram equalization is used to enhance the contrast and clarity of the images so that the vessel structures become more prominent. Finally, gamma correction is used to perform illumination correction on the images to reduce the influence caused by non-uniform illumination. In addition, random cropping is adopted to augment the training set to compensate for the relatively small number of images, so as to improve model performance and alleviate the overfitting problem under limited data conditions. By randomly selecting the center points of patches, the original images are divided into several small blocks or patches of specified sizes and different positions, and then training is performed on these patches, enabling the model to more easily learn features at different positions rather than only the specific features of the entire image. This approach helps to improve the generalization ability of the model, allowing it to perform better on new data, as shown in Figure 5.

## 4 Experimental detail and results analysis

### 4.1 Training strategy and implementation details

The experiments were conducted on a workstation equipped with 256 GB RAM, a 16-core Intel Xeon Gold 5218 CPU, and an NVIDIA Tesla T4 GPU. The model was developed using PyTorch within the PyCharm environment on Windows Server 2019, with Anaconda 3.0 and CUDA Toolkit 11.1. During training, the datasets were divided into 10,000 randomly sampled patches of size 64 × 64. The Adam optimizer was used with a learning rate of 1 × 10^−4^, and the network was trained for 50 epochs with a batch configuration of eight iterations per update cycle. For testing, patches of size 96 × 96 were extracted with a stride of 32, and the final output was generated by averaging the overlapping regions of adjacent patches to account for the inherent overlap introduced during patch-based slicing.

### 4.2 Experimental analysis

**Figure 6:**
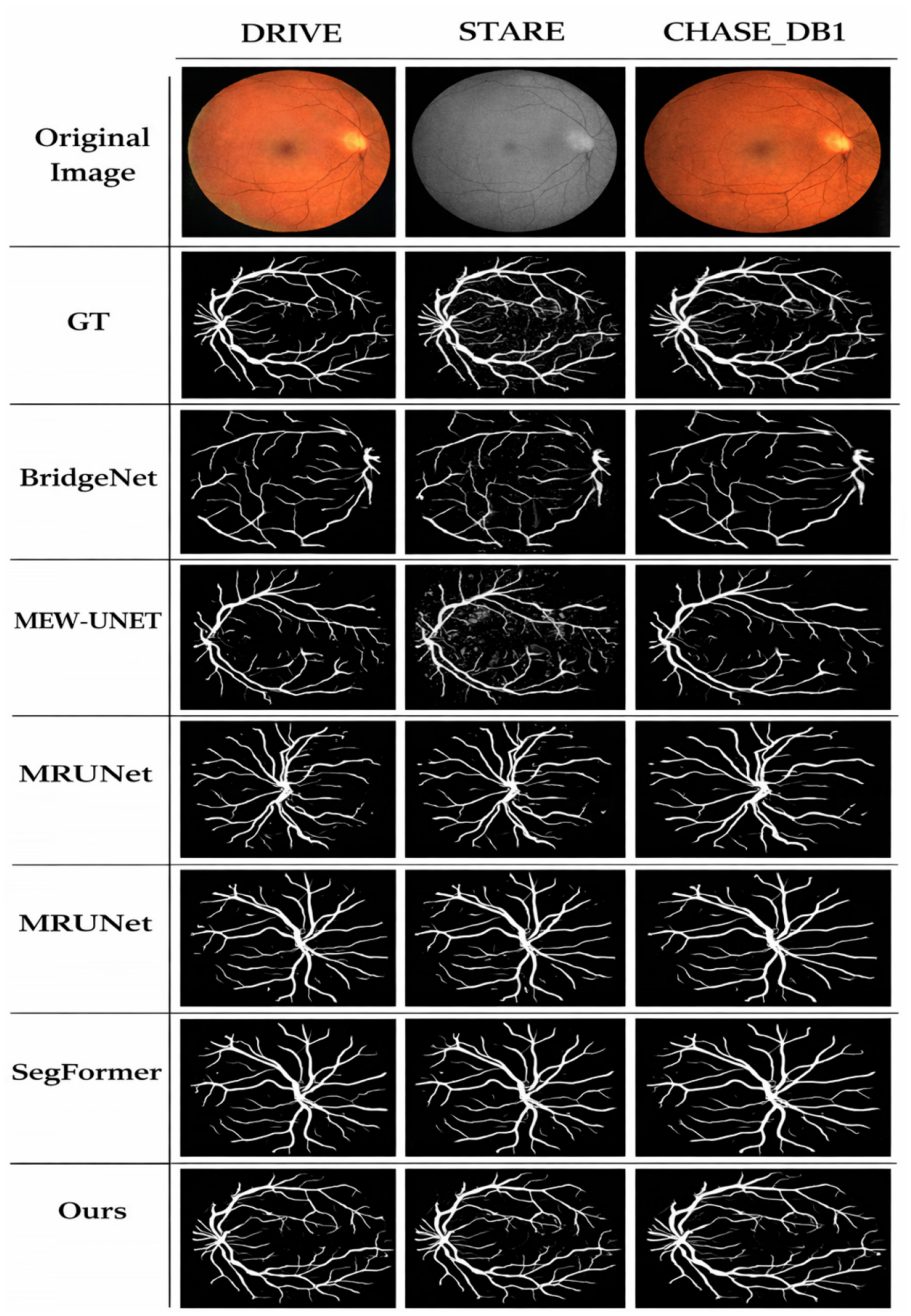
Segmentation results on different datasets.

In order to prove that the recently suggested model or algorithm is better than other algorithms in retinal vascular segmentation, we compare it to existing algorithms and perform both qualitative and quantitative evaluations. All trials are conducted in the same controlled environment to ensure a more accurate comparison. At the qualitative evaluation stage, test findings from the datasets are used to visually compare segmentation results of healthy and sick fundus images. The results of the comparative methods' segmentation on several datasets of fundus retinal vessels are shown in Figure 6. The DRIVE dataset contains images of healthy and diseased fundus retinal vessels in the first two rows, the STARE dataset contains images of healthy and diseased fundus retinal vessels in the third and fourth rows, and the CHASE_DB1 dataset contains images of fundus retinal vessels in the last two rows. Bridge-Net, MEW-UNet, MRUNet, MALUNet, and SegFormer's segmentation results are shown from left to right, with the original image and ground-truth annotation on the left. On the DRIVE, STARE, and CHASE_DB1 datasets, the quantitative evaluation results of various approaches are displayed in Tables 2–4.

**Table 2:** Quantitative comparison of retinal vessel segmentation performance on the DRIVE dataset.

Metric	ACMs	COSFIRE	Iterative	LAD-OS	CE-Net	KiU-Net	MALUNet	Bridge-Net	LadderNet	DUNet	Our (proposed)
Method type	Unsupervised				Supervised						
Accuracy	0.9510	0.9448	0.9501	0.9482	0.9524	0.9573	0.9448	0.9571	0.9568	0.9572	**0.9601**
Sensitivity	0.7445	0.7680	0.7420	0.7765	0.7840	0.7850	0.7720	0.7870	0.7875	0.7985	**0.7110**
Specificity	0.9825	0.9710	0.9790	0.9730	0.9772	0.9826	0.9700	0.9823	0.9816	0.9806	**0.9887**
AUC	0.8650	0.9630	0.9685	0.9650	0.9725	0.9815	0.9628	0.9848	0.9805	0.9816	**0.9825**
F1-score	–	–	–	–	0.8070	0.8230	0.7800	0.8220	0.8225	0.8250	**0.7840**

Bold values represent the performance achieved by the proposed method in this study.

**Table 3:** Quantitative comparison of retinal vessel segmentation performance on the STARE dataset.

Metric	COSFIRE	Iterative	LAD-OS	CPFNet	CE-Net	KiU-Net	MALUNet	MRUNet	UNet++	U-Net	Our (proposed)
Method type	Unsupervised			Supervised							
Accuracy	0.9502	0.9566	0.9560	0.9579	0.9569	0.9555	0.9230	0.9580	0.9582	0.9590	**0.9601**
Sensitivity	0.7730	0.7340	0.7810	0.6905	0.6910	0.6770	0.5910	0.6890	0.6960	0.6830	**0.7110**
Specificity	0.9708	0.9848	0.9764	0.9887	0.9874	0.9876	0.9612	0.9889	0.9883	0.9904	**0.9887**
AUC	0.9575	0.9686	0.9760	0.9769	0.9722	0.9770	0.9158	0.9710	0.9786	0.9800	**0.9825**
F1-score	–	–	–	0.7695	0.7658	0.7560	0.6115	0.7692	0.7720	0.7718	**0.7840**

Bold values represent the performance achieved by the proposed method in this study.

**Table 4:** Quantitative comparison of retinal vessel segmentation performance on the CHASE DB1 dataset.

Metric	COSFIRE	Iterative	LAD-OS	MALUNet	MEW-UNet	DCU-Net	LadderNet	DUNet	LANet	AA-UNet	Our (proposed)
Method type	Unsupervised			Supervised							
Accuracy	0.9395	0.9474	0.9460	0.9548	0.9672	0.9670	0.9662	0.9617	0.9661	0.9614	**0.9683**
Sensitivity	0.7600	0.7630	0.7640	0.7170	0.8290	0.8090	0.7995	0.8170	0.7985	0.8190	**0.7970**
Specificity	0.9593	0.9581	0.9668	0.9841	0.9843	0.9847	0.9824	0.9759	0.9829	0.9711	**0.9897**
AUC	0.9502	0.9638	0.9620	0.9769	0.9721	0.9885	0.9852	0.9817	0.9863	0.9878	**0.9899**
F1-score	–	–	–	0.7695	0.7660	0.8290	0.8045	0.7898	0.8088	0.7906	**0.8460**

Bold values represent the performance achieved by the proposed method in this study.

(1) Tests on the DRIVE Dataset: From the segmentation results of healthy fundus retinal vessels, it can be seen that MEW-UNet and MALUNet fail to effectively capture and process the features of tiny vessels at branch terminals, resulting in poor vessel connectivity. The vessel trajectories of Bridge-Net and MRUNet are relatively blurred. Unlike healthy fundus images, due to changes in tissue structure, early lesions may lead to changes in light reflection characteristics, which appear in fundus images as non-uniform brightness or changes in reflection. In particular, the boundary regions of fundus images appear darker, and the difference between vessels and surrounding pixels is sharply reduced, greatly increasing the difficulty of vessel segmentation. For example, MEW-UNet and MALUNet introduce a large amount of noise and breakages of tiny vessels when segmenting early lesion fundus images. In contrast, the proposed method exhibits strong robustness and clarity in processing both major vessels and tiny vessels in healthy and diseased retinal vessel images, and effectively suppresses the interference caused by background noise.

The segmentation outcomes of several approaches on the DRIVE dataset are quantitatively analyzed in Table 2. The results show that various fundus retinal vascular segmentation methods have variable levels of performance (Table 2). Among the unsupervised algorithms, ACMs achieves the highest accuracy at 0.9504, followed by COSFIRE at 0.9442, Iterative at 0.9494, and LAD-OS at 0.9476. These unsupervised approaches also vary in terms of specificity, sensitivity, and area under the curve (AUC). Supervised approaches outperform their unsupervised counterparts across a variety of assessment criteria when compared side by side. One possible explanation is that supervised algorithms benefit from training with huge amounts of labeled data, which allows them to better learn useful features. Even though Bridge-Net and LANet have better AUC and Spe scores than the suggested technique, it outperforms them on all other metrics, suggesting that it achieves better overall performance.

**Figure 7:**
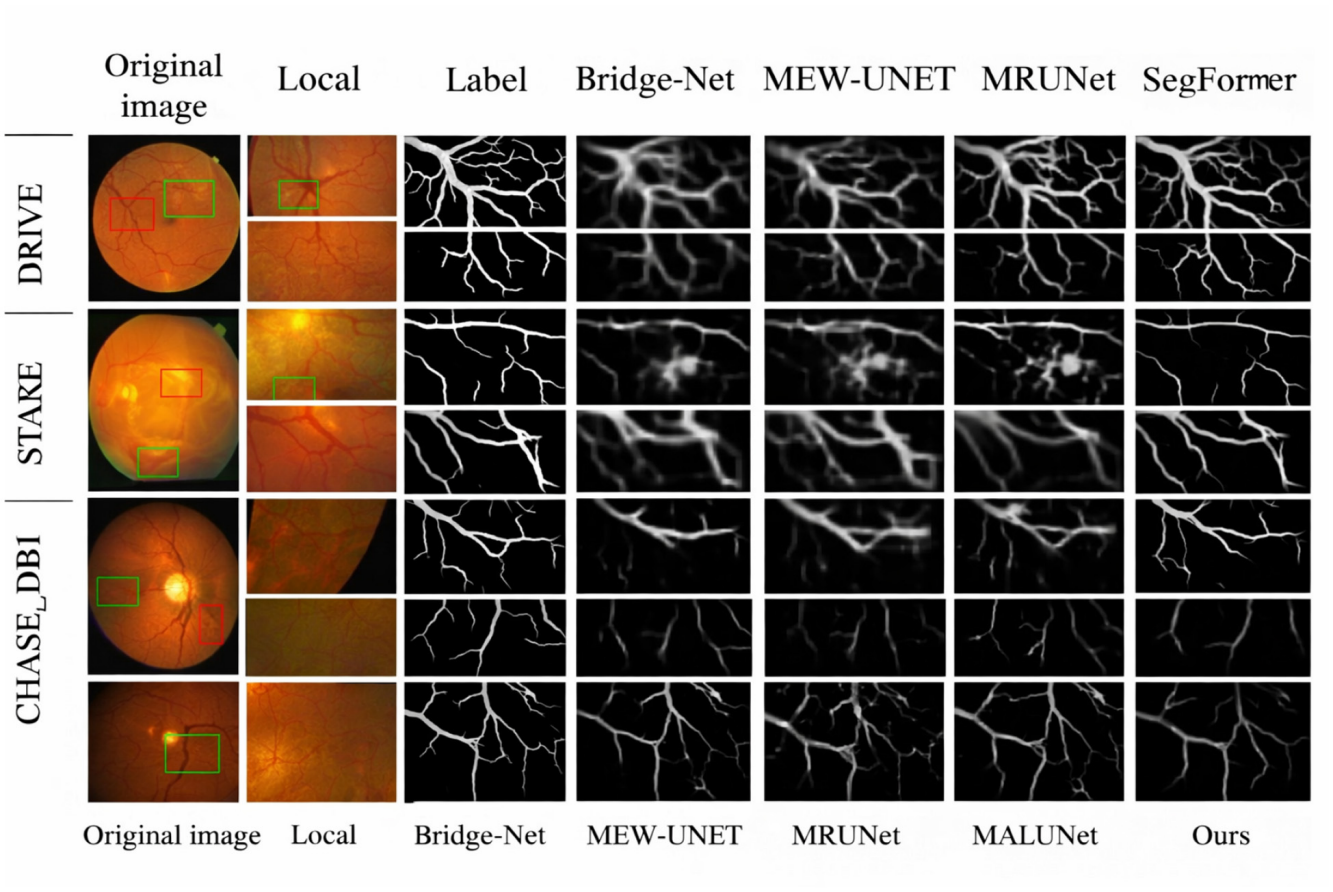
Local details of different methods.

(2) Experimental Results and Comparison on the STARE and CHASE_DB1 Datasets: The third and fourth rows in Figure 7 correspond to the retinal vessel segmentation results on healthy and diseased fundus images in the STARE dataset, and the last two rows show the retinal vessel segmentation results of different methods on the CHASE_DB1 dataset. For healthy fundus images in the STARE dataset, MALUNet obtains the worst segmentation results, and MEW-UNet cannot maintain the topological structure between vessels, resulting in breakpoints. In addition, when thick vessels within the optic disc region have branches and crossings, Bridge-Net, MRUNet, and SegFormer fail to clearly determine vessel boundaries in segmentation and obtain rough vessel edges. From the diseased fundus images, it can be seen that MEW-UNet and MALUNet are easily affected by pathological information during segmentation, resulting in a high number of false positives. In contrast, the proposed network and SegFormer produce relatively complete segmentation results for tiny vessels at branch terminals and can effectively filter out the interference of pathological information. For the CHASE_DB1 dataset, although the segmentation results of the proposed method contain

a small amount of the optic disc boundary region, it can effectively establish connections at branch crossings, and is smoother and more accurate than other methods. In addition, it can clearly segment vessels in fundus vessel boundary-blurred regions. Based on the above analysis, the proposed SGB-Net can accurately and completely segment both major vessels and thin micro-vessels, and exhibits strong robustness on both healthy and complex diseased images. To more intuitively observe the differences between different methods in local detail information, Figure 6 provides the local detail segmentation results of different methods on the DRIVE, STARE, and CHASE_DB1 datasets. Among them, the first and second columns are the original images and local enlarged regions, and the third to ninth columns are the local vessel segmentation results of the label, Bridge-Net, MEW-UNet, MRUNet, MALUNet, SegFormer, and the proposed algorithm, respectively.

### 4.3 Ablation study

**Figure 8:**
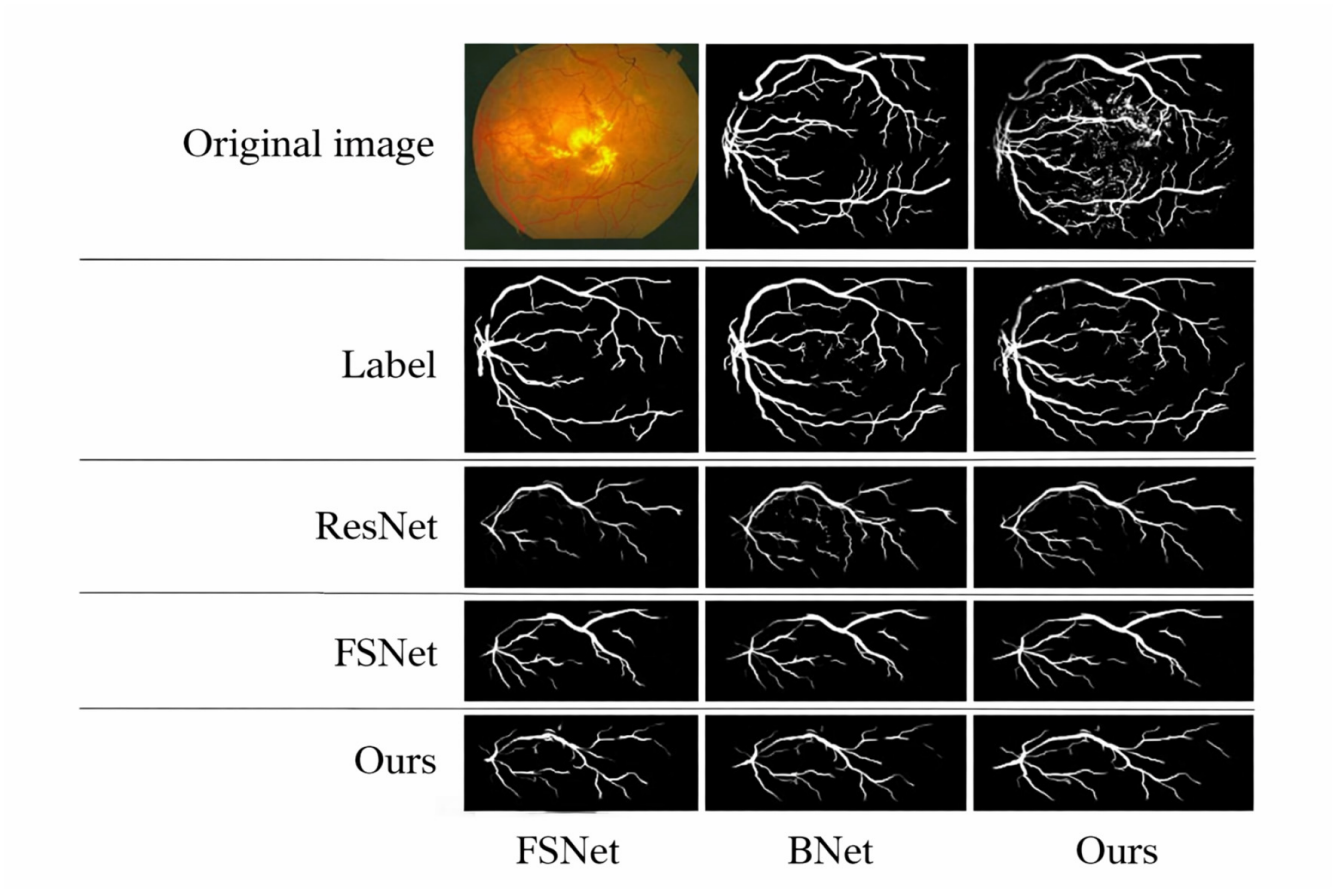
Visual comparison of different modules on the retinal vessel dataset.

Using ResNet34 as a baseline, this part performs the following ablation experiments on three retinal vascular datasets to assess the effectiveness of each module in the proposed SGB-Net. The goal is to check the effectiveness of each component. The default initialization values are used in all ablation studies to ensure a fair comparison. Figure 8 also includes quantitative scoring results.

(1) ResNet34 is used as the backbone network (denoted as ResNet);
(2) On the basis of the backbone network, the boundary refinement module is added and used as the boundary network (denoted as BNet);
(3) On the basis of the backbone network, the scale-adaptive module is added (denoted as ResNet wSA);
(4) On the basis of the backbone network, the attention enhancement module is added (denoted as ResNet wAE);
(5) On the basis of the backbone network, both the scale-adaptive and attention enhancement modules are added as the feature-guided network (denoted as FSNet);
(6) On the basis of the feature-guided network, the boundary refinement module is further added, that is, the proposed SGB-Net.

**Figure 9:**
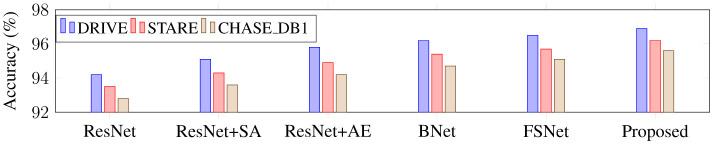
Accuracy comparison of different network modules on three retinal vessel datasets.

**Figure 10:**
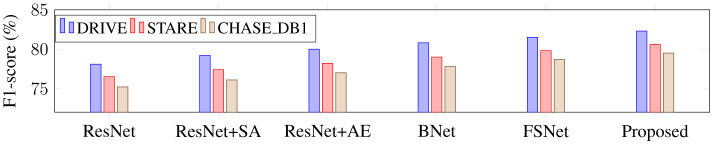
F1-score comparison of different network modules on retinal vessel segmentation.

**Figure 11:**
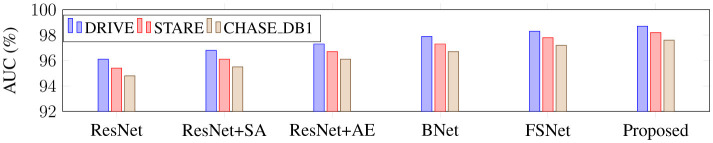
AUC performance comparison across different network modules.

**Figure 12:**
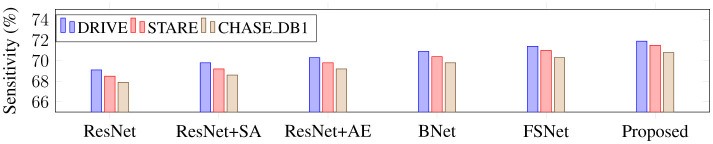
Sensitivity comparison of different network architectures on retinal datasets.

From Figure 8, it can be observed that the ResNet baseline achieves relatively poor performance scores on the three retinal fundus datasets, indicating that the performance of the backbone network is limited in vessel segmentation. This is mainly because the network cannot effectively capture local and global features in fundus images, and is easily affected by illumination and noise during the segmentation process, resulting in over-segmentation or under-segmentation. BNet adds the boundary refinement module on the basis of ResNet, and the segmentation performance is greatly improved, indicating that the boundary refinement module can distinguish boundary feature differences with similar appearances but different labels, accurately determine vessel boundaries, and thus improve segmentation accuracy. In addition, the performance obtained by both BNet and FSNet is inferior to that of the proposed SGB-Net, indicating that local detail features and contextual abstract features are crucial for vessel segmentation, and that establishing complementary connections can effectively improve network performance. Since ResNet wSA adds the scale-adaptive module on the basis of the backbone network, it can better adapt to changes in branch vessels of different sizes, and achieves better performance than the backbone network. In addition, FSNet can bring more performance improvement than BNet, because it can not only significantly learn more valuable features from complex backgrounds, thereby filtering out irrelevant features such as noise and lesion information, but also compensate for the information loss caused by the segmentation of tiny vessels. Compared with FSNet networks that contain only the SA or AE modules, the performance improvement of BNet is obvious. Compared with other variant networks, the proposed SGB-Net achieves the highest performance scores in terms of F1-score, Acc, AUC, and Sen, making the network focus more on vessel regions and avoid the interference of pathology and noise around vessels, which also demonstrates the superiority of the network structure. In addition, a sample with a complex scene and pathological information is selected for visualization comparison to more clearly show the advantages of each component in the proposed model in the segmentation task, as shown in Figures 9–12. From the figure, it can be observed that the backbone network ResNet cannot completely segment the vessel regions. Although FSNet can effectively suppress complex background and perceive changes in vessel structures, the vessel trajectories are relatively blurred and the vessel boundaries cannot be clearly determined. In contrast, the vessel boundaries captured by BNet are clearer but introduce pathological information. Since the proposed method fully utilizes the advantages of BNet and FSNet, the segmented vessels are more accurate and complete, and the interference of complex background and pathological information is effectively suppressed.

## 5 Conclusion

This study presented a retinal vessel segmentation framework that combines a progressive boundary refinement strategy with a feature-guided network incorporating scale-adaptive and attention-based modules. The boundary refinement component strengthens vessel edge representation by integrating low-level detail cues with high-level semantic features, while the feature-guided network improves the extraction of multi-scale vessel structures and reduces the influence of noise and pathological artifacts. By fusing the outputs of both branches, the proposed method achieves improved segmentation of fine vessels, better structural continuity, and enhanced boundary clarity across three public datasets. Although the framework demonstrates strong performance, several limitations remain. The model relies on patch-based training, which may restrict global contextual awareness for images with large anatomical variability. Additionally, performance may degrade in cases with severe lesions or extreme illumination changes, where vessel continuity becomes highly irregular. The current design also focuses on 2D fundus images and does not address cross-modality adaptation or real-time deployment constraints. Future work will explore three main directions: (1) incorporating global contextual modeling to strengthen the representation of long-range vessel dependencies; (2) extending the approach to broader modalities such as OCT or multimodal retinal data; and (3) optimizing computational efficiency for real-time clinical integration and large-scale screening systems. These enhancements may further improve the robustness and applicability of retinal vessel analysis in diverse clinical environments.

## Data Availability

The original contributions presented in the study are included in the article/supplementary material, further inquiries can be directed to the corresponding author.
