# Correction: AI-driven saliency-guided retinal vessel segmentation framework for sustainable digital pathology

**DOI:** 10.3389/fmed.2026.1911256

**Published:** 2026-06-30

**Authors:** Rajib Guha Thakurta, Mohammed E. Seno, Sami Ahmed Haider, Marwah A. Halwani, Supriya Ashok Bhosale, Mukesh Soni, Masood Ur Rehman

**Affiliations:** 1 School of Computer Science and Applications, REVA University, Bangalore, India; 2 Department of Computer Sciences, College of Sciences, University of Al Maarif, Ramadi, Iraq; 3 Electrical Electronics and Computer Engineer Department, School of Engineering and Physical Sciences, Heriot Watt University, Edinburgh, United Kingdom; 4 Management Information Systems Department, College of Busniess, King Abdulaziz University, Jeddah, Saudi Arabia; 5 Department of Artificial Intelligence, Vishwakarma University, Kondhwa, Pune, India; 6 Division of Research and Development, Lovely Professional University, Phagwara, India; 7 Centre for Research Impact and Outcome, Chitkara University Institute of Engineering and Technology, Chitkara University, Rajpura, Punjab, India; 8 James Watt School of Engineering, University of Glasgow, Glasgow, United Kingdom

**Keywords:** AI-driven sustainable healthcare, boundary refinement, digital pathology, retinal vessel image segmentation, saliency guidance, scale adaptively

The order of authors in the author list of the published paper was erroneously given as:

“Rajib Guha Thakurta, Mohammed E. Seno, Masood Ur Rehman, Sami Ahmed Haider, Marwah A. Halwani, Supriya Ashok Bhosale and Mukesh Soni”

The correct author list reads:

“Rajib Guha Thakurta, Mohammed E. Seno, Sami Ahmed Haider, Marwah A. Halwani, Supriya Ashok Bhosale, Mukesh Soni and Masood Ur Rehman”

The funder EPSRC, IAA Grant Number 325175 to Masood Ur Rehman was erroneously omitted. The correct **Funding** statement reads:

“The author(s) declared that financial support was received for this work and/or its publication. This work was supported by EPSRC, IAA Grant Number 325175 to Masood Ur Rehman.”

The original version of this article has been updated.

